# Melanopsin-Based Brightness Discrimination in Mice and Humans

**DOI:** 10.1016/j.cub.2012.04.039

**Published:** 2012-06-19

**Authors:** Timothy M. Brown, Sei-ichi Tsujimura, Annette E. Allen, Jonathan Wynne, Robert Bedford, Graham Vickery, Anthony Vugler, Robert J. Lucas

**Affiliations:** 1 Faculty of Life Sciences, University of Manchester, Manchester M13 9PT, UK; 2 Department of Information Science and Biomedical Engineering, Kagoshima University, Kagoshima 890-0065, Japan; 3 Department of Ocular Biology and Therapeutics, University College London Institute of Ophthalmology, London EC1V 9EL, UK

## Abstract

Photoreception in the mammalian retina is not restricted to rods and cones but extends to a small number of intrinsically photoreceptive retinal ganglion cells (ipRGCs), expressing the photopigment melanopsin [1–4]. ipRGCs are known to support various accessory visual functions including circadian photoentrainment and pupillary reflexes. However, despite anatomical and physiological evidence that they contribute to the thalamocortical visual projection [5–7], no aspect of visual discrimination has been shown to rely upon ipRGCs. Based on their currently known roles, we hypothesized that ipRGCs may contribute to distinguishing brightness. This percept is related to an object's luminance—a photometric measure of light intensity relevant for cone photoreceptors. However, the perceived brightness of different sources is not always predicted by their respective luminance [8–12]. Here, we used parallel behavioral and electrophysiological experiments to first show that melanopsin contributes to brightness discrimination in both retinally degenerate and fully sighted mice. We continued to use comparable paradigms in psychophysical experiments to provide evidence for a similar role in healthy human subjects. These data represent the first direct evidence that an aspect of visual discrimination in normally sighted subjects can be supported by inner retinal photoreceptors.

## Highlights

► Melanopsin photoreception can be selectively modulated in an intact retina ► Melanopsin supports brightness discrimination in retinal degenerate and intact mice ► Melanopsin activation induces brightness percepts in healthy humans

## Results

### Behavioral Assays of Brightness Discrimination in Mice

**Figure 1:**
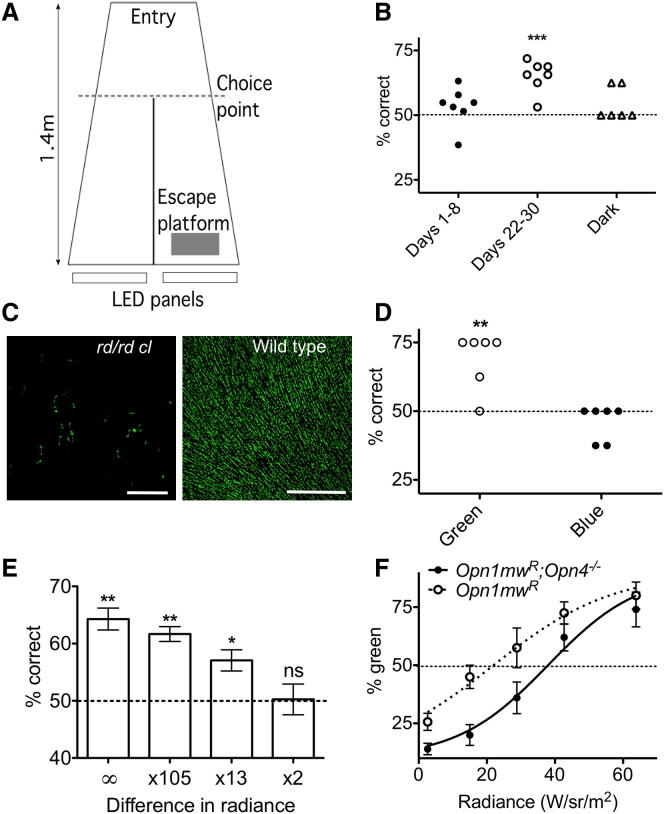
Melanopsin-Dependent Brightness Discrimination in Mice (A) Schematic of swim maze viewed from above. Visual targets (arrays of 64 blue, green, and red LEDs Figure S1) appear at the end of two lanes created by a dividing wall. An escape platform (shaded box) could be associated with a visual cue and the animal's ability to learn this association quantified by the frequency with which it chose the correct lane when first passing the end of the dividing wall (choice point). (B) The performance of *rd/rd cl* mice under training to swim toward a lit (10^6^ melanopic cd/m2) versus dark target, was not significantly better than chance over the first 8 days of testing (filled circles; p > 0.05; two-tailed one sample t test; 6–8 trials per day) but improved over repeated training to be significantly better than chance over days 22–30 (open circles, p < 0.001). Performance with the light occluded (triangles) is shown for comparison. Data are percentage of correct choices over 48 trials for each of seven mice. (C) Immunohistochemical analysis of retinal whole mounts from these *rd/rd cl* mice revealed a number of remodeled cones immunoreactive for S-opsin (green) in the ventral retina (Figure S1 for further data and methods). An equivalent image from a wild-type (WT) retina is shown for comparison. Scale bars represent 200 μm. (D) Maze navigation was not dependent upon these surviving S-cones because, although this ability was retained when a “green” stimulus (peak emission 517 nm; half peak bandwidth 30 nm) replaced the white light, their performance was no better than chance under a “blue” light providing an equivalent excitation of S-opsin (see Figure S1 for spectral radiance). (E) *rd/rd cl* mice could also be trained to identify the escape platform with the brighter of two lit targets, with the percentage of correct choice over 6 days (8 trails per day; n = 4 mice) related to their difference in radiance. Mice performed significantly better than chance (one sample t test; ^∗∗^p < 0.01, ^∗^p < 0.05) when asked to distinguish a moderately lit target (10^4^ melanopic cd/m^2^) from darkness (difference in irradiance = ∞) or targets 105 or 13 (but not 2) times brighter. (F) The frequency with which *Opn1mw^R^* and *Opn1mw^R^;Opn4^−/−^* mice previously trained to associate the escape platform with a brighter target chose a “green” lane in preference to a “red” lane is plotted as a function of the green target's radiance. Data show mean ± SEM; n = 4 for *Opn1mw^R^* and 5 for *Opn1mw^R^;Opn4^−/−^* mice; fitted with sigmoidal curves; curves differed in the predicted radiance for a 50% green choice between genotypes (F statistic; p < 0.0001) indicating a melanopsin influence on the spectral sensitivity of brightness discrimination.

One prediction of the hypothesis that intrinsically photoreceptive retinal ganglion cells (ipRGCs) contribute to assessing brightness is that this aspect of visual discrimination should survive complete loss of outer retinal photoreceptors. ipRGCs remain functional and can support a variety of accessory visual responses in mice lacking rods and cones [13]. To determine whether brightness discrimination also survives under such conditions, we used a murine model of advanced retinal degeneration (*rd/rd cl*) [14]. These mice are homozygous for a loss-of-function mutation (rd^1^) in the rod-specific cyclic guanosine monophosphate (cGMP) phosphodiesterase, which abolishes rod phototransduction and leads to rod and subsequent cone degeneration. They carry an additional diphtheria toxin-based transgene targeting surviving cones for cytoxic lesion (*cl*). At the ages employed here, *rd/rd cl* mice are essentially rodless and coneless [14]. We tested their ability to make visual discriminations in a trapezoid Y water maze in which the mouse was trained to swim toward a lit target (in preference to a dark target in the adjoining lane) to reach an escape platform (Figure 1A). Mice with an intact visual system learned to perform this task rapidly and with few errors (≥94% correct over the first 4 days of testing for each of four mice). By contrast, *rd/rd cl* animals initially appeared confused, choosing the lit lane no more often than expected by chance over 8 days of training (Figure 1B). However, over 21 days of repeated training, there was a gradual improvement in performance. At the end of this period, these animals showed significant preference for the lit target (Figure 1B).

Residual visual discrimination in *rd/rd* mice has previously been correlated with surviving cone photoreceptors, even at very late stages of degeneration [15]. Based upon previous analyses [14], we were confident that the *rd/rd cl* mice used in our maze experiments would lack cones expressing M-opsin but concerned that a few S-opsin-expressing cones might survive (these predictions were born out in subsequent immunohistochemical analyses; Figure 1C; see also Figure S1 available online). We therefore set out to test the possibility that surviving S-cones allowed brightness discrimination in this cohort of *rd/rd cl* mice. To this end, we adjusted the spectral composition and intensity of the light-emitting diode (LED) array to produce stimuli enriched for either short or longer wavelengths that should appear equally bright (isoluminant) for S-opsin (calculated according to the method in [16]). Under these circumstances, the longer wavelength stimulus was estimated to provide ∼30× greater excitation of melanopsin. We found that these *rd/rd cl* mice, previously trained to choose a lit over a dark target, were able to navigate the maze when the lit target was replaced with the longer but not the shorter wavelength light (Figure 1D). This finding excludes surviving S-cones, any other UV sensitive pigment [17], or some nonvisual output of the array (e.g., heat) as explanations for their maze navigation ability.

These experiments confirm that *rd/rd cl* mice can detect a visual signal and employ it for purposes of spatial navigation. To determine whether they could distinguish quantitative differences in brightness, we trained a new cohort of four *rd/rd cl* mice over 17 days to swim toward a lit target of moderate radiance (64 red+green+blue LED triplets each with radiance 13 W/sr/m^2^ or 10^4^ melanopic cd/m^2^) in preference to a dark target. Over the last 5 days of this training period, the mice swam toward the lit target more often than expected by chance (65% ± 2% correct, mean ± SEM; p < 0.01 one sample t test), confirming that this moderate target radiance was within the melanopsin sensitivity range. The dark target was then replaced with a target of equivalent spectral composition, but 100× higher radiance. When the escape platform was associated with this brighter target, the *rd/rd cl* mice readily learned to swim toward it. This ability was maintained when the difference in target radiance was reduced to ×13 (Figure 1E).

The performance of *rd/rd cl* mice in the water maze is consistent with the hypothesis that ipRGCs contribute to brightness discrimination. However, given the possibility of compensatory reorganization following this aggressive retinal degeneration, we were particularly interested to determine whether melanopsin also contributes to visual discrimination in animals with an intact visual system. To this end, we set out to determine whether melanopsin influences the spectral sensitivity of brightness perception in mice. Using the same swim maze paradigm employed for the *rd/rd cl* experiments, we initially trained mice to associate the escape platform with the appearance of a “green” target (64 green LEDs; 300 W/sr/m^2^ each) in preference to the null lane, which had a “red” target (64 red LEDs; 953 W/sr/m^2^ each). For this purpose, we used *Opn1mw^R^* mice that carry a knockin of the human red cone pigment (L-opsin) at the mouse M-cone opsin locus, causing cones that ordinarily would express M-opsin to instead express the human pigment [18]. Whereas the mouse M-opsin has a rather similar spectral sensitivity to melanopsin, L-opsin is shifted to longer wavelengths [19]. During the training phase, although the radiance of the red target was greater than that of the green, the reduced sensitivity of all photopigments (including the introduced L-opsin) at the longer wavelengths meant that the green target was calculated to appear “brighter” irrespective of whether the mice were basing their decision on the activity of cones, rods, or melanopsin. Accordingly, both *Opn1mw^R^* mice and *Opn1mw^R^* mice lacking melanopsin (*Opn1mw^R^;Opn4^−/−^*) rapidly learnt this task. Because mouse S-opsin is very insensitive to either red or green wavelengths [20], we felt it most unlikely that the mice were using color to discriminate between the two lanes. Nevertheless, to confirm that their choice was based on assessments of brightness, we replaced the green light with a (4×) dimmer red light. Mice of both genotypes reliably swam toward the higher radiance panel without any further training (mean >80% correct over 4–8 trials for each genotype).

We then set out to determine the radiance at which the green target appeared indistinguishable from the red, as indicated by a loss of preference for the green lane. We reasoned that, because melanopsin is practically insensitive to the longer wavelength, if it were involved in brightness assessments, this point of equal brightness should occur at lower radiances of the green light in melanopsin-sufficient versus melanopsin-knockout mice. For this work, four out of six swims per day “reinforced” the brighter preference (original array settings with platform under green target; all mice maintained >85% correct choice in this condition throughout experiment) with the remaining two being “probes” in which the platform was removed and the radiance of the green array changed. The frequency with which each mouse chose the green array on “probe” runs was recorded and expressed as a function of green radiance (Figure 1F). In both genotypes, the strong tendency to choose the green channel was lost as its radiance decreased, until eventually mice chose the red channel, indicating that they perceived it as “brighter.” Because the green array was in the melanopsin sensitivity range even at the lowest setting (10^4^ melanopic cd/m^2^; see data above for *rd/rd cl* mice), this implies that *Opn1mw^R^* mice were not relying solely on melanopsin to navigate the maze. However, the point of equal brightness, at which the mice showed no preference for either lane, occurred at lower green radiance for *Opn1mw^R^* than for *Opn1mw^R^;Opn4^−/−^* mice, indicating a melanopsin-dependent shift in spectral sensitivity.

### Using Metamers to Study Melanopsin in Mice

The change in spectral sensitivity of brightness discrimination in melanopsin knockout mice is consistent with an ipRGC contribution to this aspect of perceptual vision. A similar role for melanopsin has been proposed to explain a positive relationship between correlated color temperature and perceived brightness observed in humans under some conditions [11, 12, 21]. Determining whether melanopsin does indeed contribute to human brightness perception requires a method of selectively modulating its activity. Here we set out to achieve this using metamers. Metamers are light stimuli that appear indistinguishable to cones (and therefore have the same color and photopic luminance) despite having different spectral power distributions. Generating metamers whose stimulation of melanopsin is quite different should then allow the effects of changing melanopsin activity in isolation to be assessed [22, 23]. One limitation to this application of metamers is that although it is possible to generate stimuli that appear indistinguishable for rods as well as cones, such stimuli differ very little in predicted melanopsin excitation because melanopsin and rod opsin have similar spectral sensitivities. Therefore, in order to maximize the melanopic excitation achievable with the metamer approach, we aimed to circumvent rod-based responses by working at background light levels sufficiently bright to saturate rods.

**Figure 2:**
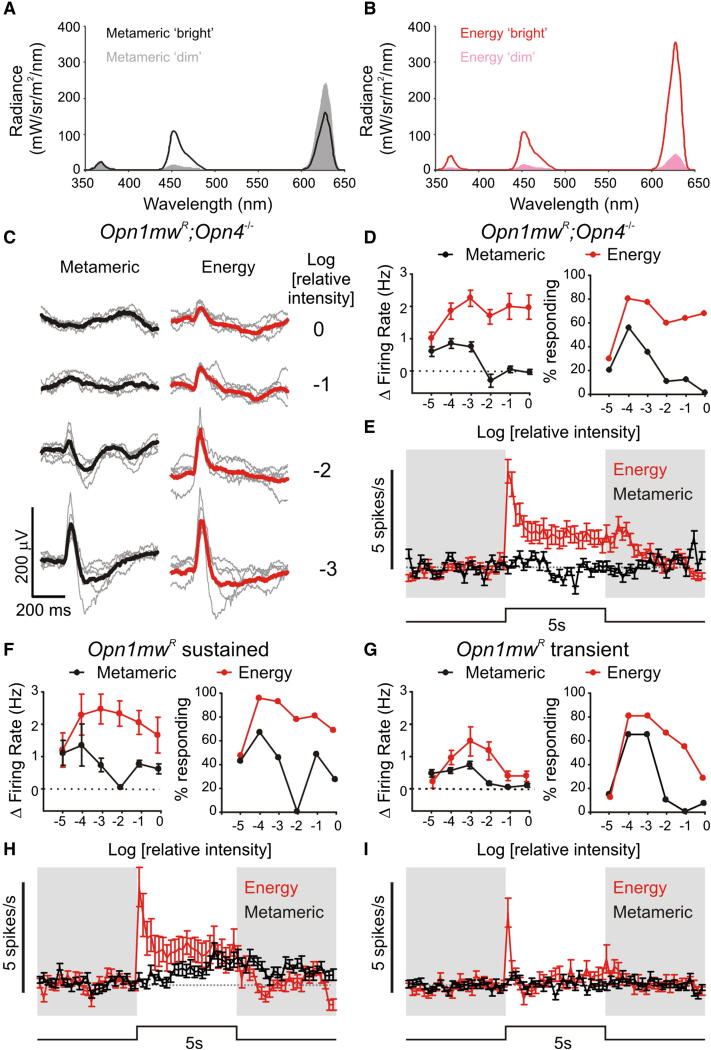
Isolating Melanopsin Responses Using Metamers (A) Spectral radiance of melanopsin “dim” and “bright” metamers. (B) Spectral radiance of energy stimuli, in which the same modulation of melanopsin as in the metameric stimuli is produced by a spectrally neutral change in radiance. (C) ERG responses in melanopsin knockout (*Opn1mw^R^*; *Opn4*^−/−^) mice evoked by “dim” to “bright” transitions of metameric (left) and energy (right) stimuli over a 1,000-fold range of irradiance. Responses to metameric stimuli disappear under photopic (rod-saturating) intensities. Thin gray traces represent individual animals (n = 5), thick traces represent mean. Numbers to right are log light intensity of the background (“dim”) condition and are expressed relative to maximum achievable (215 Scot Cd/m^2^; 700 Scot Td; 683 Melanopic cd/m^2^). (D) *Opn1mw^R^*; *Opn4*^−/−^ LGN neuronal responses to metameric and energy stimuli; (left) mean ± SEM change in firing rate following a 5 s step from “dim” to “bright” (n = 73), (right) percentage of cells with a significant increase in firing during “bright” stimuli. (E) Mean ± SEM change in firing rate of *Opn1mw^R^*; *Opn4*^−/−^ LGN neurons to metameric and energy stimuli at the highest background (Log relative intensity = 0). (F–I) Responses of WT (*Opn1mw^R^*), sustained (F and H) (n = 33), and transient (G and I) (n = 42) populations (see Experimental Procedures) to metameric and energy stimuli, quantified as in (D) and (E).

To confirm that this metamer strategy can be used to isolate melanopsin responses, we set out to establish their use in mice, in which the availability of melanopsin knockout animals [24] represents an important control. We designed a three primary (LED) system to generate a pair of metamers, isoluminant for the mouse S- and human L-opsins that account for cone photoreception in *Opn1mw^R^* mice (Figure 2A). These stimuli differed substantially in their ability to excite melanopsin, with an 8.5-fold difference in melanopic radiance between “melanopsin bright” (5,821 melanopic cd/m^2^) and “melanopsin dim” conditions (683 melanopic cd/m^2^; Figure 2A). To confirm that these stimuli truly were indistinguishable for cones, we first recorded electroretinogram (ERG) responses to transitions between the two stimuli in *Opn1mw^R^;Opn4^−/−^* mice. The prediction that these melanopsin-deficient mice should lack a retinal response to this event under rod saturating conditions was supported, with ERGs lacking at high light levels (Figure 2C). These data therefore support the view that the two stimuli are metameric for cones, and that, despite the ability of rods to function at surprisingly high light levels [25], it is possible to define conditions under which rod-based responses are undetectable.

Given the scarcity of ipRGCs, there is not a strong expectation that stimuli selectively activating this photoreceptor class would produce a measurable ERG. To confirm that these metamers elicited a melanopsin-based response, we therefore investigated electrophysiological responses in the mouse lateral geniculate nucleus (LGN) (Figures 2D–2I). A substantial proportion of visually responsive units in the mouse visual thalamus are influenced by ipRGCs [6] and should increase firing in response to melanopsin “dim” to “bright” transitions. We first confirmed that such responses were absent in the LGN of *Opn1mw^R^;Opn4^−/−^* mice. In this genotype, a large proportion of LGN units showed changes in firing associated with transitions between the metamers at low-moderate irradiances, but these were lost at higher, rod saturating, light levels. This did not reflect a general inability to elicit visual responses at such high light levels because simple increases in light intensity (“energy”; Figure 2B) drove reproducible ERG responses and elicited strong responses in the LGN under all conditions.

In mice, LGN units can be separated into “sustained” or “transient” populations based upon the degree to which they maintain increased firing over the course of an extended light pulse [6, 26]. Melanopsin's influence extends to the “sustained” but not “transient” populations [6]. Accordingly, we found that transitions from melanopsin “dim” to “bright” metamers elicited strong responses in the transient *Opn1mw^R^* LGN population only at low-moderate irradiances. By contrast, the sustained population showed responses at even the highest irradiances tested. The lack of response in the transient population and in melanopsin knockout mice under such conditions indicates that these responses originate with melanopsin. In agreement with that conclusion we found that such responses shared the poor temporal resolution reported for melanopsin phototransduction [27, 28], building up slowly over the course of the 5 s exposure to the “melanopsin bright” condition and relaxing gradually following return to the other metamer.

### Brightness Assessments in Humans

**Figure 3:**
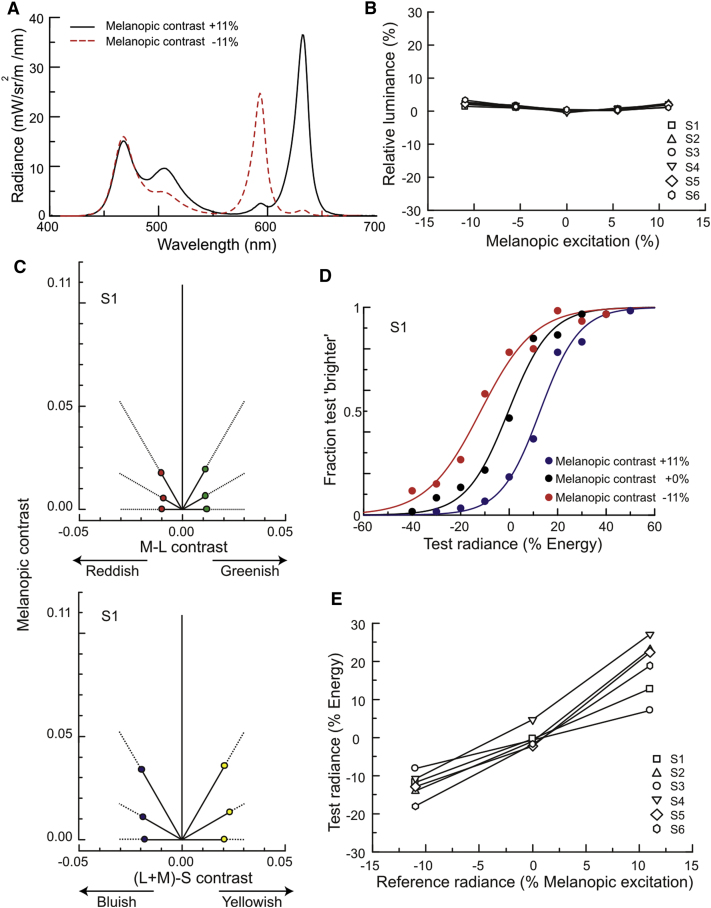
Brightness Discrimination in Healthy Human Subjects (A) The spectral radiance of metamers with the most divergent melanopic excitation generated by our four primary LED system. (B) The photopic luminance, quantified by 30 Hz heterochromatic flicker photometry, was unrelated to melanopic excitation for a range of metamers (p > 0.05 that slope of line defined by linear regression different from 0). Melanopic excitation and relative luminance are expressed as percentage with respect to data for the 0% melanopic condition for each of six subjects. (C) Color discrimination data for subject S1, showing vectors (solid line) for stimuli varying in melanopic and either M − L (top) or (L + M) − S excitation. Dots depict the threshold for detecting color change. There is no dot on the 90° vector direction because subjects did not report a color change even with the biggest change possible in this direction (11% increase in melanopic excitation). (D) The proportion of trials (out of 60) at which a representative subject (S1) reported a test stimulus (whose spectral composition matched that of the melanopic 0% stimulus) as brighter than three metameric reference stimuli (melanopic radiance +11%, 0%, −11%) is shown as a function of test radiance (shown as a percentage of change in energy with respect to test [melanopsin 0%] stimulus). The radiance at which the reference appeared indistinguishable from the test could be estimated by solving logistic functions fitted to these data (solid lines), y = {1 + exp[−(x−b)a^−1^]}^−1^, where b estimates test radiance at which the proportion of brighter responses = 0.5. (E) Across six subjects, there was a strong correlation between the test radiance at equal brightness and the melanopic excitation of the reference stimulus (one-way repeated-measures ANOVA; p < 0.001). That effect is unrelated to any impact of melanopsin on pupil size (Figure S2).

The mouse experiments thus confirm that metamers can be used to selectively modulate melanopsin. We set out to apply this approach in healthy human volunteers. A four primary LED system produced stimuli differing in predicted melanopsin but not S, M, or L cone excitation (Figure 3A). We applied these stimuli at light levels (>3,556 scot. Trolands) previously shown to saturate human rods [29] and consistent with conditions under which we were able to isolate melanopsin responses in mice (Figure 2). At these settings, the system could generate metamers whose melanopic radiance ranged from 2,234 to 2,760 melanopic cd/m^2^. The relative melanopic radiance of these stimuli are described hereafter in terms of Weber contrast, anchored to the 2,497 melanopic cd/m^2^ stimulus (0% melanopsin contrast) such that the available stimuli ranged from −11% to +11% melanopic excitation.

Standard psychophysical tests were first used to confirm that these stimuli were indistinguishable for rods and cones. In the first case, heterochromatic 30 Hz flicker photometry was used to assess their luminance. Melanopsin is unable to track such high frequency modulations, and this paradigm isolates cone-dependent assessments of brightness [30]. Accordingly, we found that subjective assessments of luminance under these conditions were unrelated to the degree of melanopsin excitation (Figure 3B).

Melanopsin contributes to gradual and sustained modulation in pupil size, whereas cone activation drives more rapid responses [22, 31, 32]. Thus, as further confirmation that transitions between metamers were silent for cones, we next showed that they elicited only sluggish changes in pupil size (Figure S2). As a final validation, we confirmed that stimuli with divergent melanopsin excitation did not differ in perceived color. For this purpose, color discrimination thresholds in both red-green (M − L) and blue-yellow [(L + M) − S] planes were calculated for stimuli differing (1) only in melanopsin excitation or (2) in both melanopsin excitation and M − L or (L + M) − S color. None of six subjects detected a difference in color when comparing among the stimuli differing only in melanopsin excitation (Figure 3C). Furthermore, the threshold for detecting a color difference between stimuli varying in either M − L or (L + M) − S dimensions was not influenced by coapplication of differences in melanopsin excitation (Figure 3C; threshold contour slopes for both color planes not significantly different from 90° across six subjects; t tests p > 0.05; mean = 90° for M − L and 88° for L + M − S dimensions). These data confirm that, over the range of melanopsin excitations achievable with our apparatus, stimuli varying in the melanopsin dimension do not differ in perceived color. This is important because perceived brightness can be influenced by chromatic saturation. Moreover, because both rods and cones contribute to color perception [33], these data provide additional confidence that these metamers are genuinely indistinguishable for conventional photoreceptors.

To determine whether differences in melanopsin excitation influence perceived brightness, we used a two-interval alternative forced-choice procedure. Subjects were asked to judge the relative brightness of three metameric stimuli (melanopic contrast −11%, 0%, and +11%) with respect to test stimuli whose spectral composition was invariant (and equivalent to the melanopsin 0% stimulus) but whose radiance changed between trials. Transitions between stimuli were set to be gradual (over 2 s), matching melanopsin's slow kinetics (Figure 2) and excluding sudden changes in irradiance to which rod and cone systems are very sensitive. The percentage of trials for which the subject identified the test stimulus as being brighter (“Tesuto sigekiga akarui” in Japanese) was then recorded for each of the three reference stimuli and over a range of radiances for the test. These data were then plotted as a function of test radiance, expressing this parameter as a percentage difference in radiance relative to the melanopsin 0% reference stimulus. In each case, the data could be fit by a logistic function (Figure 3D; Figure S2). As expected, the melanopsin 0% reference stimulus appeared indistinguishable (50% “brighter”) from the test when the latter's radiance was ∼0%. By contrast, in every individual tested (Figure 3E; n = 6), this equal brightness point occurred at lower test radiances for comparisons against the melanopsin −11% condition and at higher radiances for the melanopsin +11% condition. These results indicate that all subjects perceived greater brightness as melanopsin excitation increased.

## Discussion

The data presented here are consistent with the hypothesis that melanopsin contributes to perceived brightness in both humans and mice. However, it is important to raise a couple of caveats to that conclusion. First, although we find that both species can use melanopsin to inform assessments of brightness, imperceptible visual qualities can elicit perceptual biases [34]. It remains possible therefore that neither species actually perceives signals arising from ipRGCs. Second, although the stimulus dimension modified in these experiments is brightness (emitted light), we have not attempted to exclude all other visual parameters as an origin for our observations. In particular, it remains possible that melanopsin's major role is in lightness detection (i.e., perceiving the reflectivity of objects in the environment) because the subjects in our experiments may have interpreted a change in the light emitted by our stimuli in these terms.

The sensory task of assessing brightness (or indeed lightness) has similarities with that of measuring environmental irradiance for the accessory visual functions with which ipRGCs have heretofore been associated. On this basis, including melanopsin in these perceptual pathways is predicted to have similar advantages to those attributed to its involvement in accessory pathways, i.e., helping to measure steady-state light intensity under photopic conditions [1–4]. Future studies will be required to test this prediction.

Because spatial information is relevant for brightness perception in a way that it may not be for, e.g., circadian photoentrainment, our data place renewed emphasis on defining the spatiotemporal resolution of melanopsin-based vision. There has been a report [7] that mice lacking rod and cone photoreception retain quite high resolution spatial discrimination (although see [35, 36]). It will be important to confirm that finding in models with more complete rod and cone loss and to determine whether melanopsin contributes such spatial information in visually intact animals. It will also be interesting to define melanopsin's contribution (if any) to other aspects of vision. Dacey et al. [5] revealed unique chromatic opponency to cone influences on primate ipRGCs suggesting that this ganglion cell class may contribute to color vision. Here, we failed to observe a color percept associated with changing melanopsin excitation, but this may reflect the small change in melanopsin activation achievable with the metamer approach.

Crude light perception survives in patients with advanced retinal degeneration, and it has previously been suggested that this could reflect melanopsin photoreception [37]. Our work with *rd/rd cl* mice confirms that it is possible for such visual discrimination to originate with melanopsin. However, the poor visual performance of such mice is consistent with the experience of patients and questions the practical importance of this phenomenon. We therefore see the most significant aspect of our work as being the evidence that melanopsin contributes to visual discrimination in normally sighted individuals. This provides a new way of thinking about currently unexplained aspects of brightness and/or lightness perception. On a practical level, it also suggests that aligning the spectral quality of light sources more closely to melanopsin could represent a new approach to improving energy efficiency and enhancing perceived brightness.

## Experimental Procedures

### Mouse Behavior

All animal procedures were carried out in accordance with UK Animals (Scientific Procedures) Act 1986. Protocols for swim maze were based upon published methods for testing visual discrimination [38]. Briefly, a plexiglass trapezoid (1.4 m long, 0.85 and 0.25 m wide) was constructed with a wall separating the wider of the ends and protruding 60 cm into the maze. LED arrays (LED-500DX/RGB; Monacar Intl, Bremen, Germany; see Figure S1 for spectral radiance) were placed at the end of each lane created by this divider. An escape platform placed at the end of one of these lanes could be hidden beneath opaque water.

Adult (>80 days) *rd/rd cl*, *Opn1mw^R^*, and *Opn1mw^R^;Opn4^−/−^* mice were first acclimated to the apparatus over at least 4 days in which they were introduced to the maze at increasing distances from the escape platform until they swam to it from the back of the maze. During subsequent training, the location of the escape platform was associated with a particular setting for the LED array. In this phase, mice were given 60 s to find the platform before being guided to it by hand. Location of the enforced target was randomly assigned throughout experiments, except that >3 successive appearances in the same lane were forbidden.

### Mouse Physiology

Electroretinograms were recorded in five adult male *Opn1mw^R^;Opn4^−/−^* mice (150–200 days) according to published methods [35] and electrophysiological responses in the LGN were recorded from urethane anesthetized adult male mice (80–160 days) as previously described [39]. Briefly, multiunit activity was recorded using a 32 ch. recording probe (A4X8-5 mm-50-200-413; Neuronexus, Ann Arbor, MI, USA) introduced to the LGN using stereotaxic coordinates (Bregma: −2.5 mm; Midline: −1.9 to −2.5; Depth: −2.6 mm relative to brain surface). Probe placement confirmed post hoc by histology. Conventional spike sorting approaches (Offline Sorter; Plexon) were used to isolate single unit activity.

Visual stimuli were delivered to mice in a darkened chamber from a custom-built source (Cairn Research, Kent, UK) consisting of independently controlled UV, blue, and red LEDs (λmax: 365, 460, and 630 nm, respectively). Light was combined by a series of dichroic mirrors and focused onto a 5 mm diameter piece of opal diffusing glass (Edmund Optics Inc., York, UK) positioned <1 mm from the eye (contralateral to the recording probe for LGN recordings). LED intensity was controlled by a PC running LabView 8.6 (National Instruments).

MATLAB (The Mathworks, Natick, MA, USA) modeling was used to identify metameric stimuli providing large differences in melanopsin activation. Spectral sensitivity functions for mouse opsins were approximated by visual pigment templates [40] with appropriate λ_max_, corrected for lens transmission [41]. Melanopic radiance was calculated as described in [42]. Figure 2 presents data from stimuli providing an 8.5-fold difference in melanopic excitation. At highest irradiance, these were calculated to produce a photon flux equivalent to 15.5 and 14.5 Log photons/cm^2^/s at wavelength of peak sensitivity for L- and S-cones, respectively, and to vary between 14.7 and 15.6 Log photons/cm^2^/s for melanopsin. Effective flux for mouse rods was calculated to vary between 14.6 and 15.5 Log photons/cm^2^/s. Responses to transitions between these metamers were compared with those to spectrally neutral increases in energy presenting an equivalent increase in melanopsin excitation while also modulating cones (14.9 to 15.8 and 13.8 to 14.7 Log photons/cm^2^/s for L- and S-cones, respectively).

At each background intensity/stimulus combination, we tested 15 × 5s steps from the “dim” to “bright” condition (interstep interval = 35 s). For LGN recordings, the ipsilateral eye was illuminated with a 460 nm LED approximately matched to the intensity of the contralateral stimuli. At the end of these experiments, mice were dark adapted for 15 min after which we evaluated responses to 5 × 60 s steps of 460 nm light applied to the contralateral eye (14.8 Log melanopsin photons/cm^2^/s) to identify sustained and transient LGN neuron populations as previously described [6].

### Human Experiments

Six visually corrected subjects (age range: 21–27 years; average 23.3 years) participated in the brightness-matching experiment, flicker photometry, and pupil measurement experiments. Five of these also participated in the color discrimination experiment and three in the brightness-matching experiment using the artificial pupil. All subjects had normal ocular health and color vision according to the Ishihara color blindness test and gave written informed consent. The study was approved by the local research ethics committee.

### Visual Stimuli

Stimuli were generated by a four-primary illumination system [22, 43] employing LEDs at peak wavelengths 635 nm, 595 nm, 510 nm, and 470 nm whose output was controlled by both pulse width modulation and an embedded controller (H8/3052; Renesas Technology, Japan). The output of these LEDs was mixed in an integrating sphere and projected to a diffusing surface to produce a circular stimulus occupying 95° of the visual field. Test stimuli were measured with a spectroradiometer (CS-1000A, KonicaMinolta, Japan) and represented in a receptor-excitation space that used excitation derived from three types of cones and melanopsin. The melanopsin 0% stimulus had a Commission Internationale de l'Eclairage (CIE) coordinate of (0.351, 0.311) and a luminance of 356 cd/m^2^.

### 30 Hz Flicker Photometry

The luminance of metameric stimuli was measured by asking subjects to adjust the luminance of a control (melanopsin 0%) stimulus to minimize the perception of flicker. Data were averaged from five sessions.

### Color Discrimination

Subjects were presented with a background comprising the melanopsin 0% stimulus and then asked to report changes in color upon presentation of 2 s test stimuli. Test stimuli varied in melanopic excitation and/or red-green or blue-yellow planes. Thresholds for seven different vector directions were measured in the same session using interleaved staircases (0°–180° in 30° steps). For this experiment, stimuli subtended 44° of the visual angle but excluded a 5° small circular field at the fovea to minimize the appearance of a small color spot, known as the Maxell spot. Melanopic contrast varied only in positive values.

### Brightness Matching

Subjects were asked to compare the brightness of pairs of stimuli (test and reference) presented against a background comprising the melanopsin 0% condition. Stimuli were presented for 1 s, and highlighted by an audible tone, transitions from the background and between stimulus pairs occurred over 2 s. Psychometric functions (Figure 2D; Figure S2) were measured with the method of constant stimuli. Each pair was presented 60 times. Subjects were instructed to indicate which of the two stimuli was brighter; they received no feedback on their responses. Recording sessions started with 5 min of adaptation to the background, followed over 35 min with presentation of multiple stimulus pairs with pseudorandomized order for test and reference stimuli. Stimuli were monocular (left eye masked). Reference stimuli were one of three metamers (melanopsin −11%, 0%, +11%) at a fixed radiance. Test stimuli comprised the melanopsin 0% condition at various radiances.
