# Supplementary material for: Melanopsin-Based Brightness Discrimination in Mice and Humans
Source: Curr Biol. 2012 Jun 19;22(12):1134–41. doi: 10.1016/j.cub.2012.04.039 (PMC3509338; doi:10.1016/j.cub.2012.04.039)
Supplement: Document S1. Figures S1 and S2 [file mmc1.pdf]

Current Biology, Volume *22*

Supplemental Information

Melanopsin-Based Brightness Discrimination in Mice and Humans

Timothy M. Brown, Sei-ichi Tsujimura, Annette E. Allen, Jonathan Wynne, Robert Bedford, Graham Vickery, Anthony Vugler, Robert J. Lucas

Supplemental Inventory

1. Supplemental Figures

Figure S1, related to Figure 1

Figure S2, related to Figure 3

2. Supplemental References

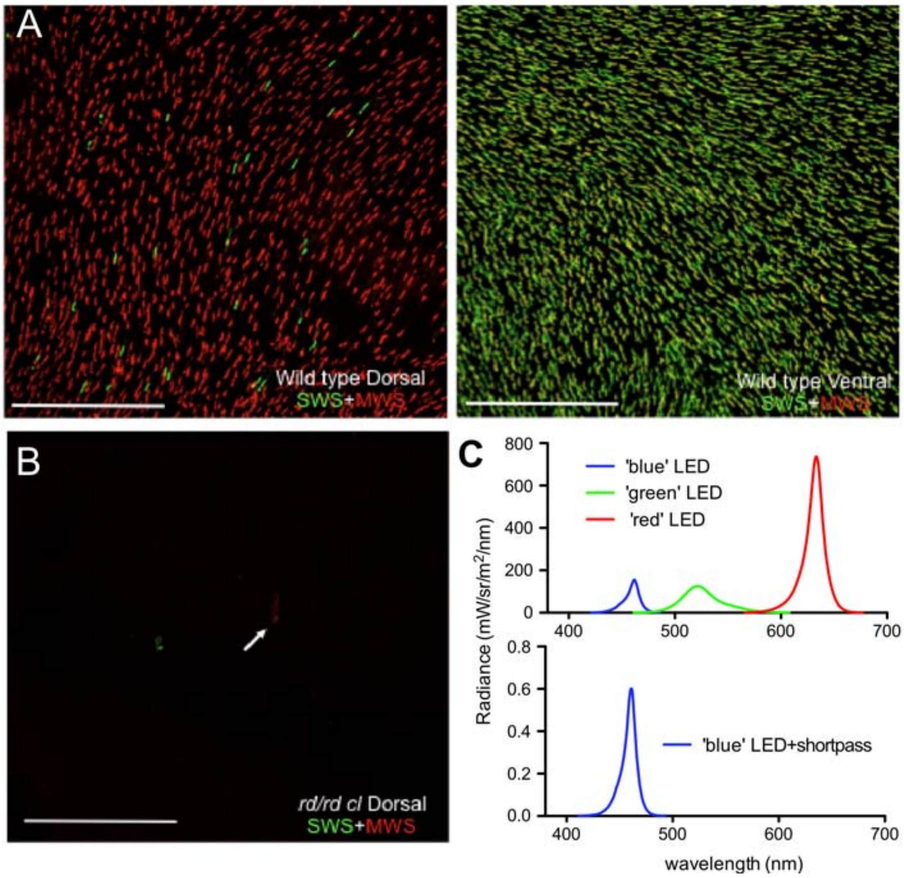

Figure S1. Cone Opsin Expression in Retinally Degerenate Mice and Spectral Radiance of LED Arrays

Upon completion of behavioural assessment, the *rd/rd cl* mice trained to navigate the swim maze (and 2 age-matched C3H wild type mice) were perfused with 4% paraformaldehyde and processed for wholemount retinal immunofluorescence double labelling as previously described [40]. The primary antibodies used to concurrently label short and long-wave length sensitive cones were goat anti-S opsin (1:500 dilution, Santa Cruz Biotechnology) and rabbit anti-M opsin (1:5000 dilution, Millipore). The total population of residual S and M cones were counted using a Leica DMRB fluorescence microscope (under X10 magnification). Representative pictures from dorsal and ventral retina were taken using a Zeiss LSM510 confocal microscope and LSM image browser software.

(A) Wild type control retinas showed the expected dorso-ventral gradient in the expression of S-opsin (green) and M-opsin (red). We found a few surviving S-opsin immunoreactive cones in the ventral retina of all *rd/rd cl* mice (Figure 1). However, all but one of the *rd/rd cl* retinas were completely denuded of M-immunoreactive cones.

(B) A single *rd/rd cl* retina had three M-opsin immunoreactive cones (arrow in B points to one of these) in the dorsal retina. Excluding data from this individual from all analyses did not change any of the conclusions described in the main body of this manuscript. Scale bars = 200µm.

(C) Visual targets for swim maze experiments comprised arrays of 192 LEDs (64 each of ‘red’, ‘green’ and ‘blue) in a 12x16 array. The spacing of these LEDs (up to 5cm between LEDs of the same colour) is predicted to be beyond the spatial resolution of mouse vision when viewed from the end of the dividing line (‘choice point’), rendering each array in effect a large diffuse target (18x30° of visual field at choice point). Top panel shows the spectral radiance of a single LED of each colour at maximum output (measured with spectroradiometer from Bentham Instruments, Reading, UK). LEDs of each colour could be switched on independently and their intensity controlled by current modulation and neutral density filters. In the experiment testing the hypothesis that *rd/rd cl* mice use a very few surviving S-cones to navigate the maze (Figure 1D), a short pass filter (V67 dichroic filter, Lee filters, Hants, UK) was applied to the ‘blue’ LED (bottom panel).

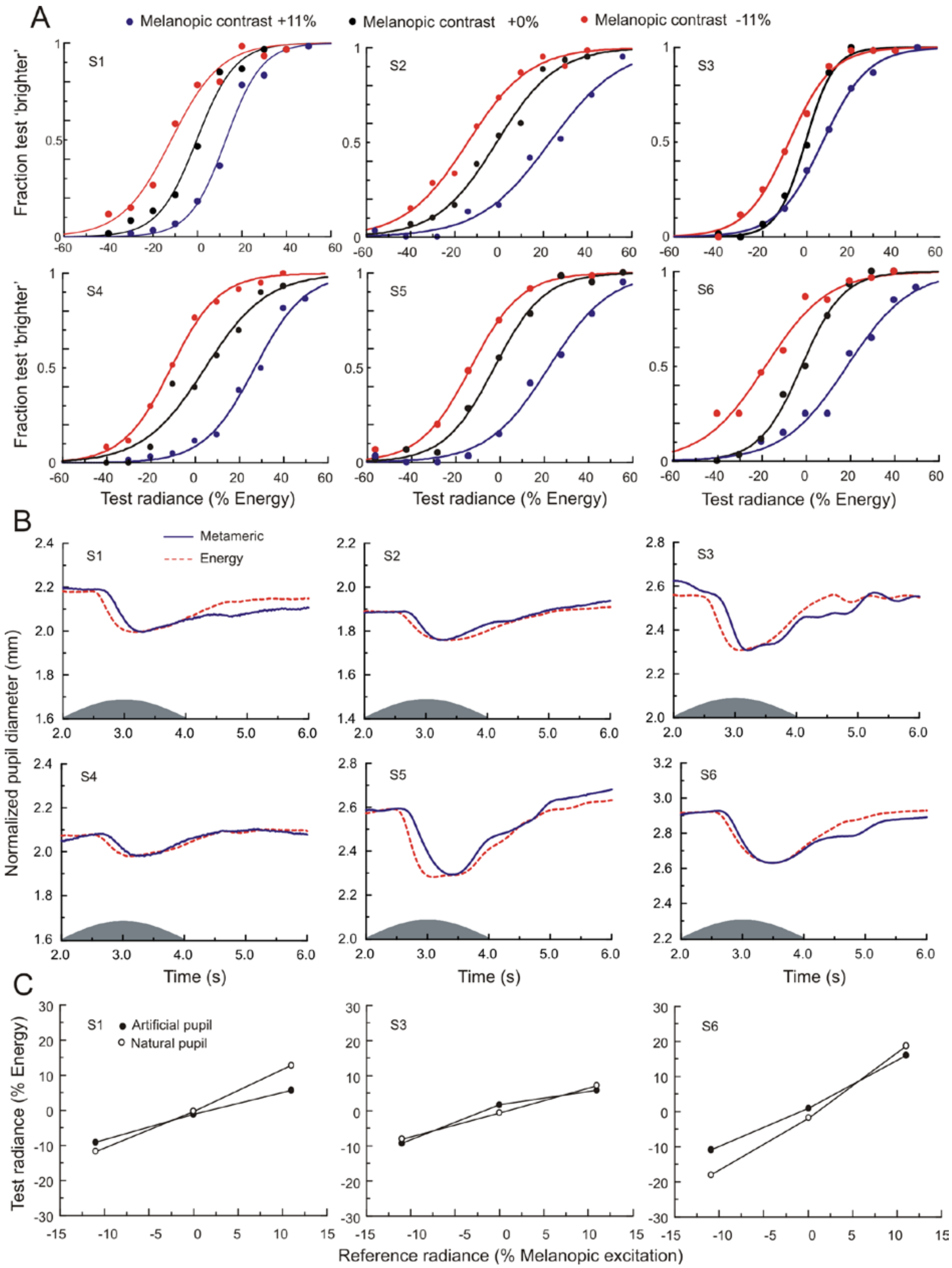
A
Melanopic contrast +11%
Melanopic contrast +0%
Melanopic contrast -11%
S1
S2
S3
S4
S5
S6
Fraction test 'brighter'
Test radiance (% Energy)
B
Metameric
Energy
Normalized pupil diameter (mm)
Time (s)
C
Artificial pupil
Natural pupil
Test radiance (% Energy)
Reference radiance (% Melanopic excitation)

Figure S2. Melanopsin Effects on Pupil Size and Brightness Discrimination

(A) The proportion of trials (out of 60) at which each subject contributing to figure 3 reported a test stimulus whose spectral composition matched that of the melanopic 0% stimulus as brighter than three metameric reference stimuli (melanopic radiance +11%, 0% -11%). Data points are plotted as a function of test radiance (shown as a % change in energy with respect to the melanopsin 0% stimulus). The radiance at which the reference appeared indistinguishable from the test could be estimated by solving logistic functions fitted to these data (solid lines), $y = \{1 + \exp[-(x-b)a^{-1}]\}^{-1}$, where b estimates test radiance at which the proportion of brighter responses = 0.5).

(B) Pupil responses (measured according to published methods [21]) of all subjects to metameric (solid line) and energy (hatched line) stimuli. Stimuli were gradual ramps up and down (timing shown in grey) presented against a background of the melanopsin 0% condition. For metameric stimuli the effective melanopsin activation was increased by 11% without a concomitant change in cone excitation. The energy stimulus comprised a 50% increase in irradiance across all wavelengths. Both stimuli induced a pupil constriction, but its latency was increased in the metameric 722±15 ms compared to the energy (552±28 ms) condition (n = 6, mean±SD, $P < 0.01$, by paired t-test). This is consistent with published evidence that melanopsin-derived pupil responses have delayed onset compared to those driven by cones [21, 30].

(C) Melanopsin-driven pupil constriction would be predicted to antagonise the positive relationship between melanopsin excitation and perceived brightness reported here (Figure 3). Nevertheless, to confirm that that effect was retained in the absence of pupil regulation, relative brightness experiments were compared in 3 subjects using an artificial pupil (solid circle) vs. a natural pupil (open circle). Data from these subjects is shown here. Brightness matching protocol, and methods of data collection and presentation are as described in the main body of the text.

Supplemental References

21. Tsujimura, S., and Tokuda, Y. (2011). Delayed response of human melanopsin retinal ganglion cells on the pupillary light reflex. Ophthalmic Physiol. Opt. 31, 469–479.

30. McDougal, D.H., and Gamlin, P.D. (2010). The influence of intrinsically-photosensitive retinal ganglion cells on the spectral sensitivity and response dynamics of the human pupillary light reflex. Vision Res. 50, 72–87.

40. Vugler, A.A., Semo, M., Joseph, A., and Jeffery, G. (2008). Survival and remodeling of melanopsin cells during retinal dystrophy. Vis. Neurosci. 25, 125–138.
